# Efficacy of an oral formulation of afoxolaner and milbemycin oxime against *Tunga penetrans* in naturally infected dogs

**DOI:** 10.1186/s13071-023-06063-x

**Published:** 2023-12-02

**Authors:** Katharine Costa dos Santos, Eric Tielemans, Andre Antonio Cutolo, Paula Elisa Brandão Guedes, Tatiani Vitor Harvey, Jamille Bispo de Carvalho Teixeira, Rebeca Costa Vitor, Anaiá da Paixão Sevá, Adan William de Melo Navarro, Ana Carolina Ribeiro Lima, Karin Denise Botteon, Thammy Vieira Bittar, George Rêgo Albuquerque, Fernando de Almeida Borges, Frederic Beugnet, Renata Santiago Alberto Carlos

**Affiliations:** 1 Departamento de Ciências Agrárias e Ambientais, Universidade Estadual de Santa Cruz (UESC), Campus Soane Nazaré de Andrade, Rod. Jorge Amado, Km 16 - Salobrinho, Ilhéus, Bahia 45662-900 Brazil; 2 Boehringer Ingelheim Animal Health, 29 Avenue Tony Garnier, 69007 Lyon, France; 3 Missouri Research Center, Boehringer Ingelheim Animal Health, 6498 Jade Rd., Fulton, MO 65251 USA; 4 College Station, USA; 5 Boehringer-Ingelheim Saúde Animal, 14171 Pça. das Nações Unidas, 18° andar (Torre B), São Paulo, SP 01449-010 Brazil; 6 Faculdade de Medicina Veterinária e Zootecnia, Universidade Federal de Mato Grosso do Sul (UFMS), Av. Sen. Filinto Müler, 2443 - Pioneiros, Campo Grande, Mato Grosso do Sul 79070-900 Brazil

**Keywords:** Afoxolaner, Dog, Efficacy, Field, *Tunga penetrans*

## Abstract

**Background:**

The sand flea *Tunga penetrans* is one of the agents of tungiasis, an important parasitic skin disease affecting humans and several mammalian species. Tungiasis is mainly observed in disadvantaged rural and peripheral urban communities in Latin America and sub-Saharan Africa. The dog is a major reservoir of *Tunga* fleas. Hematophagous adult female *Tunga* spp. embed and grow in their host’s epidermis and cause cutaneous inflammatory disorders. NexGard Spectra^®^ is an orally administered endectocide for dogs, a co-formulation of the isoxazoline afoxolaner and the macrocyclic lactone milbemycin oxime. The objective of this study was to assess the efficacy of this product against canine tungiasis.

**Methods:**

A blinded, negative-controlled field trial was conducted in a Brazilian community known to be highly endemic for tungiasis. Sixty-six dogs naturally infected with live *T. penetrans* were randomly allocated to a treated group (44 dogs) and an untreated control group (22 dogs). In a first phase, dogs from the treated group were treated on days 0, 30, and 60. Efficacy was evaluated on the basis of the macroscopic parasitic skin lesions (Fortaleza classification) on days 7, 14, 21, 30, 45, 60, 75, and 90. In a second phase, to evaluate natural reinfections, all dogs were treated on day 90 and evaluated every 2 weeks thereafter until at least 30% of dogs were infected with live sand fleas.

**Results:**

During the first phase, efficacy (reduction in live sand fleas) of 92.4% was demonstrated on day 7. From day 14 until day 90, the efficacy of NexGard Spectra^®^ was 100%. In the second phase, all dogs were free of live *T. penetrans* from 15 until 45 days after the day 90 treatment; 60 days post-treatment, 11% of dogs were reinfected, and 75 days post-treatment, 40% of dogs were reinfected.

**Conclusions:**

NexGard Spectra^®^ was demonstrated to be highly effective against canine tungiasis. In addition to an obvious beneficial effect on the health and welfare of the treated dog, the use of this product may have a one-health benefit on human cases by controlling the main reservoir of sand fleas.

**Graphical Abstract:**

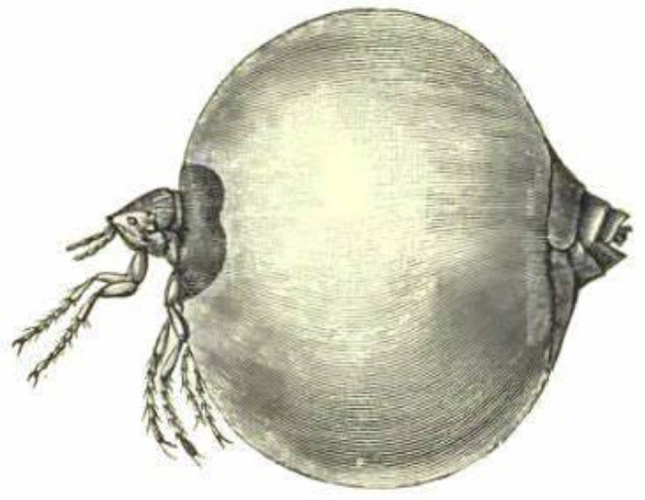

## Background

 Sand fleas of the genus *Tunga* (Siphonaptera, Tungidae, Tunginae), also called chigoe fleas and jigger fleas, are among the agents of tungiasis, a zoonotic parasitic skin disease [1]. Tungiasis is an important and severe public health concern in tropical and subtropical regions such as Latin America, the Caribbean, and sub-Saharan Africa [2]. Tungiasis is included in the list of neglected tropical diseases by the World Health Organization, in the category of scabies and parasitic skin diseases [3]. High human prevalence can occur in disadvantaged rural and peripheral urban communities [4–8].

The 1-mm-long adult *Tunga* spp. inhabit the floor and infest a mammalian host mainly by direct skin contact. They usually affect feet in humans, pads in animals, or other skin areas with frequent floor contact. After minutes to hours of host contact, the hematophagous sand flea takes a blood meal, and within a few hours, the female penetrates the epidermis, remains embedded, and matures into a globular pea-size neosome growing by abdominal hypertrophy. The neosomy period lasts approximately 5–6 weeks before parasite death, and causes a highly morbid and intense inflammatory reaction, increasing in intensity at the late stage [9–13]. When the epidermis penetration phase is complete, during stage III of the Fortaleza classification, (Table 1), hundreds of eggs are released into the environment by each female. Similar to other Siphonaptera, the eggs hatch into larvae that feed on organic debris and molt into pupae that contribute to efficient and lasting environmental contamination in sand, soil, dust, dirt, and cracks in floors [5].

**Table 1:** Description of the Fortaleza classification

Stage	Characteristics
I	Penetration phase (30 min to a few hours). Reddish spot of approximately 1 mm
II	Hypertrophy beginning (1–2 days post-penetration). Dark spot of approximately 1–2 mm usually in the middle of a hyperemic area
III	Hypertrophy maximum (2 days–3 weeks post-penetration). Round glassy yellow spot, often raised, with a central dark spot about 4–13 mm in diameter
IV	Dead parasite (3–5 weeks post-penetration). Brown to black raised circular patches surrounded by necrotic tissue
V	Residual scar (6 weeks to several months post-penetration). A shallow circular crater in the skin with necrotic edges

Fourteen *Tunga* species are known, including the zoonotic species *Tunga penetrans*, the most prevalent and widely described sand flea species in Brazil [14]. *Tunga penetrans* frequently infests humans and domestic, semi-domestic, and wildlife mammals such as dogs, cats, pigs, cattle, goats, and rats [14–18]. Endemicity in humans is exacerbated in poor communities where the standards of medical care and hygiene are low, and where there is close proximity to domestic, semi-domestic, or stray reservoir animals. [1, 2, 19]. Dogs in Brazil and pigs in Africa are considered the main reservoirs [19, 20]. A prevalence of infection as high as 86% has been described in Brazilian dogs in highly endemic areas [21, 22]. The incidence of human tungiasis can reach 50% in highly endemic Brazilian areas [23]. Besides environmental infection by the off-host developing stage, inter-host transmission of adult *Tunga* is hypothesized to occur in crowded areas [5]. Some cases have been detected in travelers returning from endemic areas [23–26] but are probably underdiagnosed, as they generally do not require specialist treatment [23].

The control strategies for tungiasis are challenging and require an integrated approach [11, 17, 27]. They include environmental sanitation and control of immature off-host stages, human treatment, and animal reservoir management including treatment and animal–human proximity control [28]. The epidemiology is also influenced by the climate, as infection peaks are observed in dry seasons [27]. In areas with consistent climatic conditions, control is further complicated as contamination is constant throughout the year.

Afoxolaner is a systemic insecticide and acaricide compound belonging to the isoxazoline group, acting on gamma-aminobutyric acid (GABA)-gated chloride ion channels, and resulting in electrophysiological disruption of the central nervous system and death of the arthropod [29, 30]. Afoxolaner is available in three oral formulations for dogs (NexGard^®^, NexGard Spectra^®^, and NexGard^®^ Plus, Boehringer Ingelheim Animal Health) and is indicated for the treatment and control of flea (*Ctenocephalides felis* and *C. canis*), tick, and mite infestations [31–33]. Afoxolaner was also demonstrated efficacious against the screwworm *Cochliomyia hominivorax* in dogs in a field trial in Brazil [34]. After oral administration, alone or in combination with milbemycin oxime (MO), afoxolaner is rapidly absorbed, with plasma peak levels (*T*_max_) observed between 2 and 6 h, and is slowly eliminated, with an elimination half-life of 15 ± 8 days, resulting in rapid onset of efficacy, and sustained efficacy lasting 1 month [35, 36]. Afoxolaner is highly bound to plasma proteins, therefore acting through a systemic pathway on hematophagous arthropods.

The efficacy of fluralaner, another isoxazoline compound, has already been demonstrated against *T. penetrans* in dogs [37].

This manuscript describes a field study designed to assess the afoxolaner insecticidal activity against *T. penetrans* in naturally infected dogs in an endemic region of Brazil.

## Methods

This study was a blinded, negative-controlled, and randomized field trial conducted in one site in Brazil from November 2021 to May 2022. It was conducted in accordance with the principles of good clinical practice (VICH GL 9).

The study site was located in a resource-poor rural community (district of Aritaguá, Ilhéus, Bahia) known to have a high tungiasis prevalence [21, 22]. The local climate is steady throughout the year, with average annual temperatures ranging from 22 to 25 °C, and with a regular and consistent rainfall regime. The village was populated with 368 residents and approximately 100 dogs. Some residences were unfinished and did not have concrete floors, and streets were unpaved and consisted of sand and earth. Dogs were semi-domestic and roamed freely, but were habituated to return to and dwell in the same house or house yard.

The investigated veterinary product (IVP) was NexGard Spectra^®^, a palatable tablet formulation for oral administration to dogs, containing afoxolaner and MO. It is recognized that MO, the nematicide compound of the product, has a negligible effect on arthropods when given orally once a month [38, 39], and thus this compound does not bring any additional ectoparasiticide efficacy to that of afoxolaner. Depending on body weight, the administered doses range from 2.5 to 5.3 mg/kg afoxolaner and 0.5–1.1 mg/kg MO. NexGard Spectra^®^ is indicated for the treatment of *Ctenocephalides felis* and *C. canis* fleas, ticks, mites, gastrointestinal nematodes, lungworms, and eyeworms, and for the prevention of heartworm disease [39]. During the study, all treatments were administered according to label instructions.

The canine tungiasis was primarily evaluated using the Fortaleza classification [12] (Table 1) and secondarily using the severity score for acute dog tungiasis (SCADT) classification (Table 2).

**Table 2:** Description of severity score for acute dog tungiasis (SCADT)

Clinical signs	Number of affected locations	Score
Hyperemia and edema	1–5	1
	6–10	2
	11–16	3
Pain at the site when pressed	1–5	1
	6–10	2
	11–16	3
Suppuration and formation of abscesses^a^	1–5	1
	6–10	2
	11–16	3
Cluster of lesions^b^	1–5	1
	6–10	2
	11–16	3
Fissure(s)^a^	1–5	1
	6–10	2
	11–16	3
Skin ulcer^a^	1–5	1
	6–10	2
	11–16	3
Mutilation of lesions regardless of the sites involved^c^		2
Altered gait/lameness		3
Ectopy of lesions		0.5^d^

The maximum individual score (SCADT) for a dog is 27 (23 + 4)

^a^Regardless of the number of foci and the size of the area involved

^b^Three or more lesions close together (1–2 mm apart)

^c^Mutilation of lesions indicating severe itching

^d^For each ectopic body part involved, up to a maximum of eight ectopic sites; maximum 4 points

At each visit, a detailed skin inspection was performed, including paws, limbs, tail, mammary glands, abdomen, testes, and nose, to search for tungiasis lesions. Before examination, the dogs’ paws were cleaned using a brush and water to improve lesion detection and scoring. Identified lesions were staged according to the Fortaleza classification. Each clinical sign was scored for each affected area, and the results added to obtain the SCADT. If an animal exceeded a SCADT score of 22, the dog was treated by surgical removal of *Tunga* spp. neosomes and removed from the study for ethical reasons, as it was not needed in this trial.

To be eligible for inclusion, dogs had to be affected with at least three tungiasis lesions of stage II or III of the Fortaleza classification [12], corresponding to the live embedded stages of the fleas.

The dogs had to comply with the NexGard Spectra^®^ label (e.g., weighing at least 2 kg, aged at least 8 weeks), and had to be healthy and have a suitable temperament. Ten neosomes were collected from 10 different dogs for morphological speciation and underwent identification in the Veterinary Parasitology Laboratory of Santa Cruz State University, Brazil. All neosomes were identified as being from *T. penetrans*.

Throughout the study, usual husbandry conditions were maintained, and commercial canine feed was provided by study personnel. At the beginning of the study, dogs from the untreated control group were administered a dewormer containing febantel, pyrantel pamoate, and praziquantel (Therax Plus, UCB Pharma) by unblinded personnel. All study activities were performed at the respective dogs’ house yard and with documented owner consent. Personnel responsible for the parasitic and clinical evaluations were blinded to study group.

Dogs were assigned to the treated group or the untreated control group on a 2:1 ratio, per order of inclusion and on the basis of a random allocation list. Each individual dog was an experimental unit, and dogs from the same house yard were assigned independently to the treated or the untreated control group.

Forty-four dogs were assigned to the IVP-treated group, and 41 completed the study. Twenty-two dogs were assigned to the untreated control group, and 21 completed the study. The four dogs that did not complete the study were removed for reasons unrelated to the study design or the IVP; all interim data obtained from these dogs were nevertheless included in the analyses. All dogs were mongrels and were identified with a subcutaneous microchip. The main characteristics of the dogs and baseline measurements are described in Table 3.

**Table 3:** Distribution of age, weight, sex, flea count, and total severity score (SCADT) on day 0

	Treated group	Control group
Number of dogs [*n*]	44	22
Age [years]: mean (median) [range]	3.8 (3) [0.25–15]	3.4 (2) [0.7–9]
Body weight [kg]: mean (median) [range]	9.5 (8) [2.6–28.2]	10.9 (10) [3.7–22.0]
Sex	14 females, 30 males	12 females, 10 males
Flea count mean of lesions on stages II and III (median)	18.8 (8.5)	19.4 (10.5)
Flea count lesions on stages II and III range	3–68	3–63
SCADT mean (median)	2.1 (2)	2.6 (2.75)
SCADT interval	0–8.5	0–4.5

All males and females were intact (none were spayed or neutered)

There was no statistical difference between the SCADT of the two groups on day 0 (*P* = 0.264)

The study was conducted in two phases. Phase I was designed to evaluate the efficacy of three monthly treatments with the IVP against tungiasis. Phase II was designed to evaluate natural reinfections after a single treatment in this specific environment.

In phase I, dogs from the treated group were administered IVP orally on days 0, 30, and 60. The control group remained untreated. Dogs were evaluated for tungiasis weekly after the first treatment and then every 2 weeks after the second and third treatments, i.e., on days (±2) 7, 14, 21, 30, 44, 60, 74, 90. In phase II, on day 90, all dogs (including the previously treated group and the control group) received an IVP treatment and were evaluated every 2 weeks until reappearance of tungiasis lesions in at least 30% of dogs. Dogs were thus evaluated on days (±2) 105, 120, 135, 150, and 165, when the 30% reinfection threshold had been exceeded.

### Data analysis

*Primary variable* The *T. penetrans* lesion counts of stages II and III of the Fortaleza classification were the primary variable, as they corresponded to the live embedded stages.

Efficacy was calculated on each evaluation time point using two methods:

- Group comparison, where the groups were compared at each post-treatment evaluation time point, using the formula:
  $${\text{Efficacy}}\,(\%)\, = \,\left( {M*{\text{ control}}--M* {\text{treated}}} \right)/\left( {M*{\text{ control}}} \right) \times {1}00$$
  *M = GM (geometric mean) of combined stage II and III lesions (Fortaleza classification). For information, efficacy based on AM (arithmetic means) was also calculated.

The data obtained were analyzed per the Shapiro–Wilk method to determine whether the distribution was parametric or non-parametric. Considering that the distribution was non-parametric, the Wilcoxon test was used.

- Comparison of the percentage of dogs infected with parasites in each group, at each post-treatment evaluation time point, using the formula:
  $${\text{Efficacy}}\,\left( \% \right) = [\left( {{\text{Nc}}--{\text{Nt}}} \right)/\left( {{\text{Nc}}} \right] \times {1}00$$
  Nc = % of animals with active lesions in the control group, Nt = % of animals with active lesion in the treated group.

The 95% confidence limits for the percentage of dogs free of live *T. penetrans* were calculated as Wilson scoring intervals.

Total severity scores (SCADT) were the secondary variable and were calculated by study day and study group.

The evaluation of the SCADT for canine tungiasis was based on the mean and median of the clinical signs on each assessment day. Mean SCADT scores in the two groups were compared at each post-treatment evaluation time point using the Wilcoxon test (exact), with the level of significance set to *α* = 0.05 (two-sided).

## Results

### Phase I: evaluation of the efficacy of three monthly treatments with NexGard Spectra^®^

#### Primary variable

The efficacy results based on Fortaleza classification II or III lesions in the treated group compared to the untreated control group are detailed in Table 4.

**Table 4:** Phase I, efficacy results based on means of Fortaleza classification II or III lesions in the IVP-treated group and the untreated control group

Day	GM^a^			AM^b^			*P*-value^c^
	IVP	Control	% Eff^d^	IVP	Control	% Eff^d^	
0	11.84	13.66	–	18.84	19.41	–	0.483
7	0.59	7.77	92.43	0.955	17.41	94.6	< 0.0001
14	0	8.58	100	0	20.05	100	< 0.0001
21	0	8.38	100	0	20.86	100	< 0.0001
30	0	5.65	100	0	13.59	100	< 0.0001
44	0	2.10	100	0	4.86	100	< 0.0001
60	0	0.93	100	0	4.86	100	< 0.0001
74	0	1.14	100	0	4.19	100	< 0.0001
90	0	1.81	100	0	9.95	100	< 0.0001

^a^Geometric mean of combined stage II and III lesions (Fortaleza classification)

^b^Arithmetic mean of combined stage II and III lesions (Fortaleza classification)

^c^Wilcoxon test (Mann–Whitney)

^d^Efficacy (%) = (GM/AM control − GM/AM treated)/(GM/AM control) × 100

On day 0 before treatment, the average baseline sum of both levels of lesion was 18.8 in the treated group (*n* = 44) and 19.4 in the untreated control group (*n* = 22). On day 7 the efficacy (percent reduction) observed in the treated group in comparison to the untreated control group was 92.4% by geometric mean (94.5% by arithmetic mean). From day 14 until day 90, the efficacy of the IVP was maintained at 100%.

The percentage of flea-free dogs in the treated group and the untreated control group are shown in Table 5.

**Table 5:** Phase I, efficacy results based on the percentage of *Tunga*-free dogs in the IVP-treated group and the untreated control group

Day	IVP group		Control group		*P*-value^c^	% Eff^d^
	*n*^a^	%^b^	*n*^a^	%^b^		
0	0 in 44	0.0	0 in 22	0.0	NA	NA
7	25 in 44	56.8	2 in 22	9.0	< 0.0001	52.5 (31.6–67.0)^b^
14	44 in 44	100	2 in 22	9.0	< 0.0001	100
21	43 in 43	100	3 in 22	13.6	< 0.0001	100
30	43 in 43	100	4 in 22	18.1	< 0.0001	100
44	43 in 43	100	10 in 21	47.6	< 0.0001	100
60	42 in 42	100	12 in 21	57.1	< 0.0001	100
74	41 in 41	100	13 in 21	61.9	< 0.0001	100
90	41 in 41	100	11 in 21	52.4	< 0.0001	100

^a^*n* = number of animals free of *Tunga* lesions in the group

^b^% = percentage of animals free of *Tunga* lesions in the group

^c^*t*-test

^d^Efficacy (%) = (Nc − Nt)/(Nc)] × 100, where Nc = % of animals with active lesions in the control group, Nt = % of animals with active lesion in the treated group

As per inclusion requirements, on day 0, 100% of dogs from each group were infected by live embedded *T. penetrans* (i.e., were affected with Fortaleza level II and/or III lesions). On day 7, 25/44 dogs (57%) in the treated group and 2/22 (9%) dogs in the untreated control group were free of live *T. penetrans*. From day 14 to day 90, all dogs in the treated group were free of live *T. penetrans*. The infection levels improved somewhat in the untreated control dogs, especially during the third month (on days 60, 75, and 90), when 52–62% of dogs were diagnosed free of live *T. penetrans,* nevertheless remaining significantly lower than the treated group over the 3 months (*P* < 0.0001 at all time points).

#### Total severity scores (SCADT)

The SCADT by study day and study group and the corresponding intervals are shown in Table 6. Mean total SCADT differed significantly between groups on days 7, 14, 21, 30, 60, and 90, whilst on days 44 and 74 there was no significant difference between groups.

**Table 6:** Phase I, SCADT severity scores per group and time point

Day	Treated group			Control group			*P*-value
	Mean ± SD	Median	Interval	Mean ± SD	Median	Interval	
0	2.1 ± 2.12	2	0–8.5	2.6 ± 2.20	2.75	0–4.5	0.264
7	0.61 ± 1.28	0	0–6	1.00 ± 1.31	0	0–4	0.121
14	0.47 ± 1.13	0	0–5	2.38 ± 2.96	1	0–11	< .0001
21	0.37 ± 0.81	0	0–3	1.77 ± 2.59	1	0–9	0.003
30	0.09 ± 0.42	0	0–2	0.95 ± 1.30	0	0–4	< .0001
44	0.16 ± 0.30	0	0–3	0.19 ± 0.80	0	0–4	0.785
60	0.09 ± 0.43	0	0–2	0.71 ± 1.60	0	0–7	0.024
74	0.21 ± 0.88	0	0–5	0.52 ± 1.03	0	0–3	0.078
90	0.00 ± 0.00	0	0–0	1.22 ± 2.40	0	0–8	< .0001

### Phase II: evaluation of natural reinfections after a single treatment

On day 90, all dogs were treated and then monitored every 2 weeks for the reappearance of active tungiasis lesions (i.e., level II or III Fortaleza classification).

The percentages of dogs that remained free of active tungiasis lesions are shown in Table 7.

**Table 7:** Phase II, percentage of *Tunga*-free dogs after a single administration of NexGard Spectra^®^ on day 90

Day	Previously IVP-treated group^a^		Previously untreated control group^a^		All dogs	
	*n*^b^	%^c^	*n*^b^	%^c^	*n*^b^	%^c^
90	41 in 41	100	11 in 21	52.4	52 in 62	84
105	41 in 41	100	21 of 21	100	62 in 62	100
120	41 in 41	100	21 of 21	100	62 in 62	100
135	41 in 41	100	21 of 21	100	62 in 62	100
150	36 in 41	87.8	19 of 21	90.5	55 in 62	89
165	22 in 41	53.65	15 of 21	76.2	37 in 62	60

^a^Previous group denomination, not adequate for phase II, as all dogs received an IVP treatment on day 90

^b^*n* = number of animals free of *Tunga* lesions in the group

^c^% = percentage of animals free of *Tunga* lesions in the group

The 10 dogs that previously belonged to the untreated control group and were affected with active tungiasis lesions before the day 90 treatment confirmed the results of phase I, as they were cleared from active lesions on day 105, 15 days after their IVP treatment. All 62 dogs remained free of new infection with *T. penetrans* until 45 days after treatment (day 135). Eleven percent of dogs were reinfected 60 days after treatment (day 150) and 40% were reinfected 75 days after treatment (day 165) when the study was closed.

In both phases, no adverse reaction related to treatment was observed in any dog.

## Discussion

The results of this field trial demonstrated a high level of efficacy of the IVP for the treatment and control of tungiasis in dogs, in a highly endemic area. Phase I demonstrated that 100% efficacy was achieved within 2 weeks after the first treatment and was maintained at 100% with monthly treatments. Phase II demonstrated that a single treatment provided sustained efficacy of 100% for at least 45 days, but that a regular treatment regimen was necessary, as environmental reinfection occurred afterwards. This study also demonstrated that the treatment significantly improved the dermal skin lesions associated with *T. penetrans* infections.

**Fig. 1:**
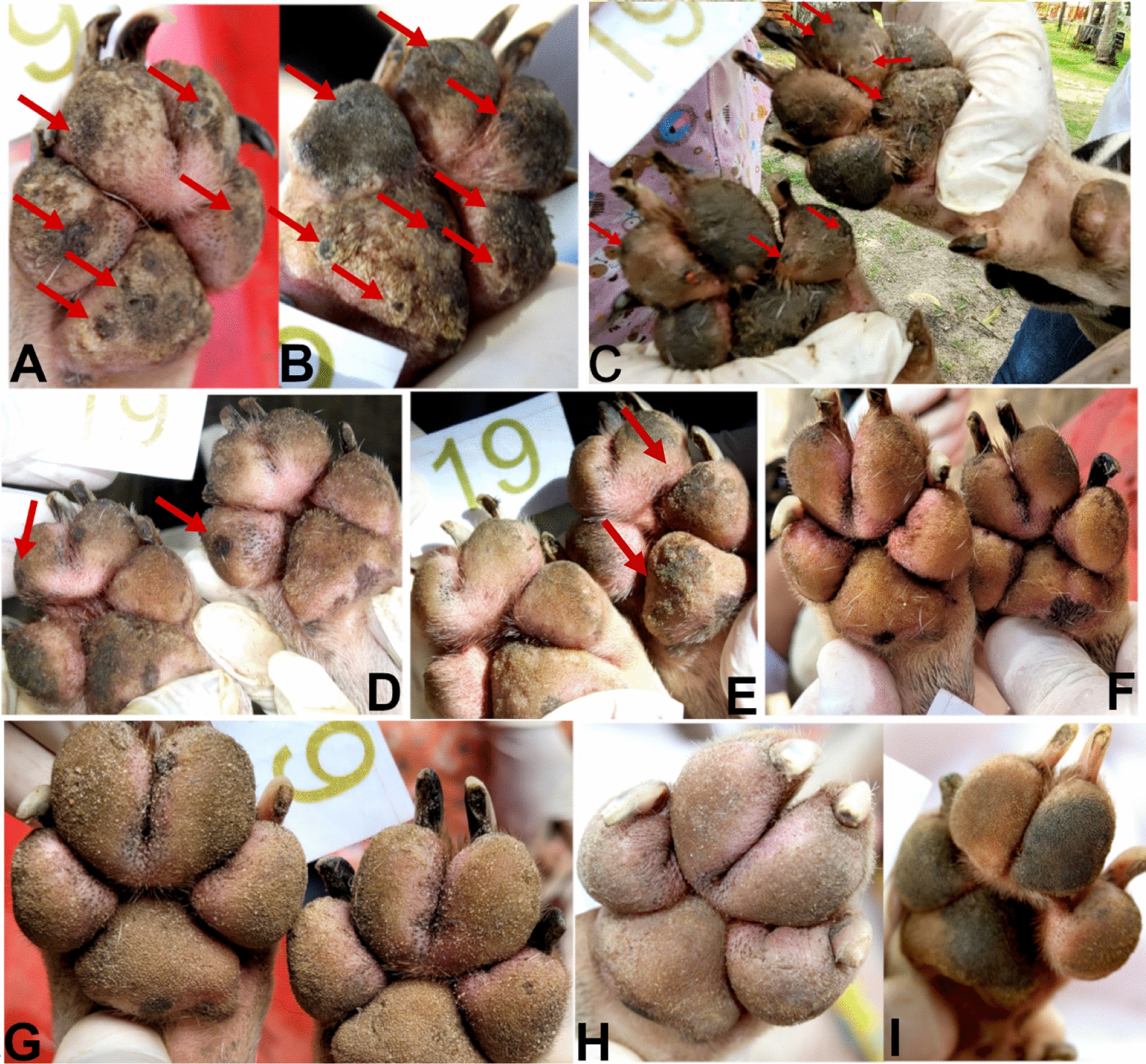
Evolution of *T. penetrans* lesions for dog 19 of the treated group (*source*: personal collection). **A**, **B** Day 0, the dog had multiple stage II and III lesions located on the front feet pads; **C** day 7, the pads had no longer stages II and III lesions, stage IV lesions were visible (arrows); **D** day 14, **E** day 21, stage IV lesions were visible (arrows); **F** day 30; **G** day 60, the pads had no longer any *T. penetrans* lesions; **H**, **I** day 90, the pads were free of *T. penetrans* lesions and completely re-epithelialized

**Fig. 2:**
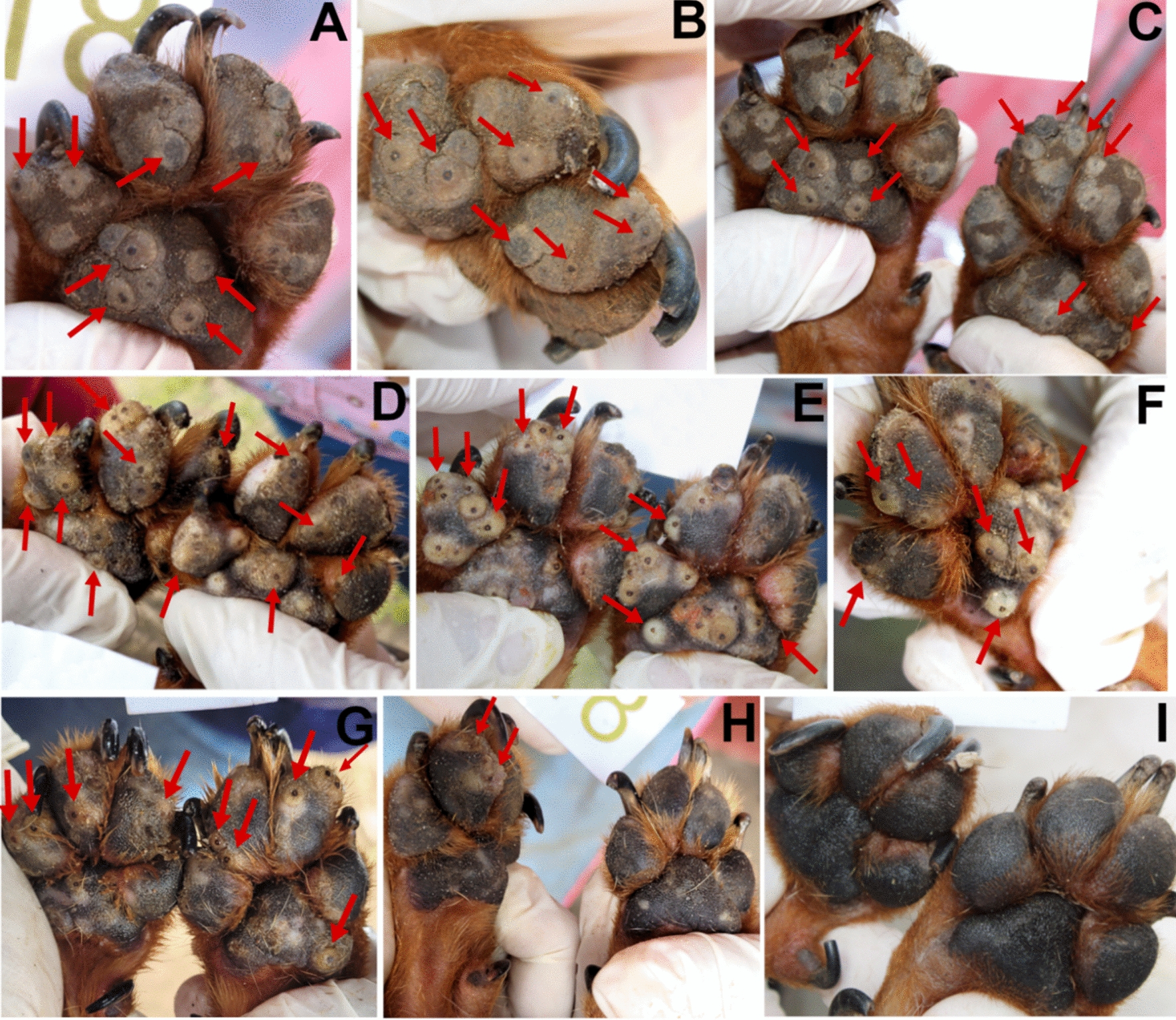
Evolution of *T. penetrans* lesions for dog 18 of the untreated control group (co-living with dog 19, Fig. 3) (*source*: personal collection). **A**–**C** Day 0; **D** day 7; **E** day 14, **F** day 21; **G** day 30; **H** day 60, the dog had multiple stage II and III lesions located on the front feet pads (arrows); **I** day 90, the pads were free of stage II and III lesions

**Fig. 3:**
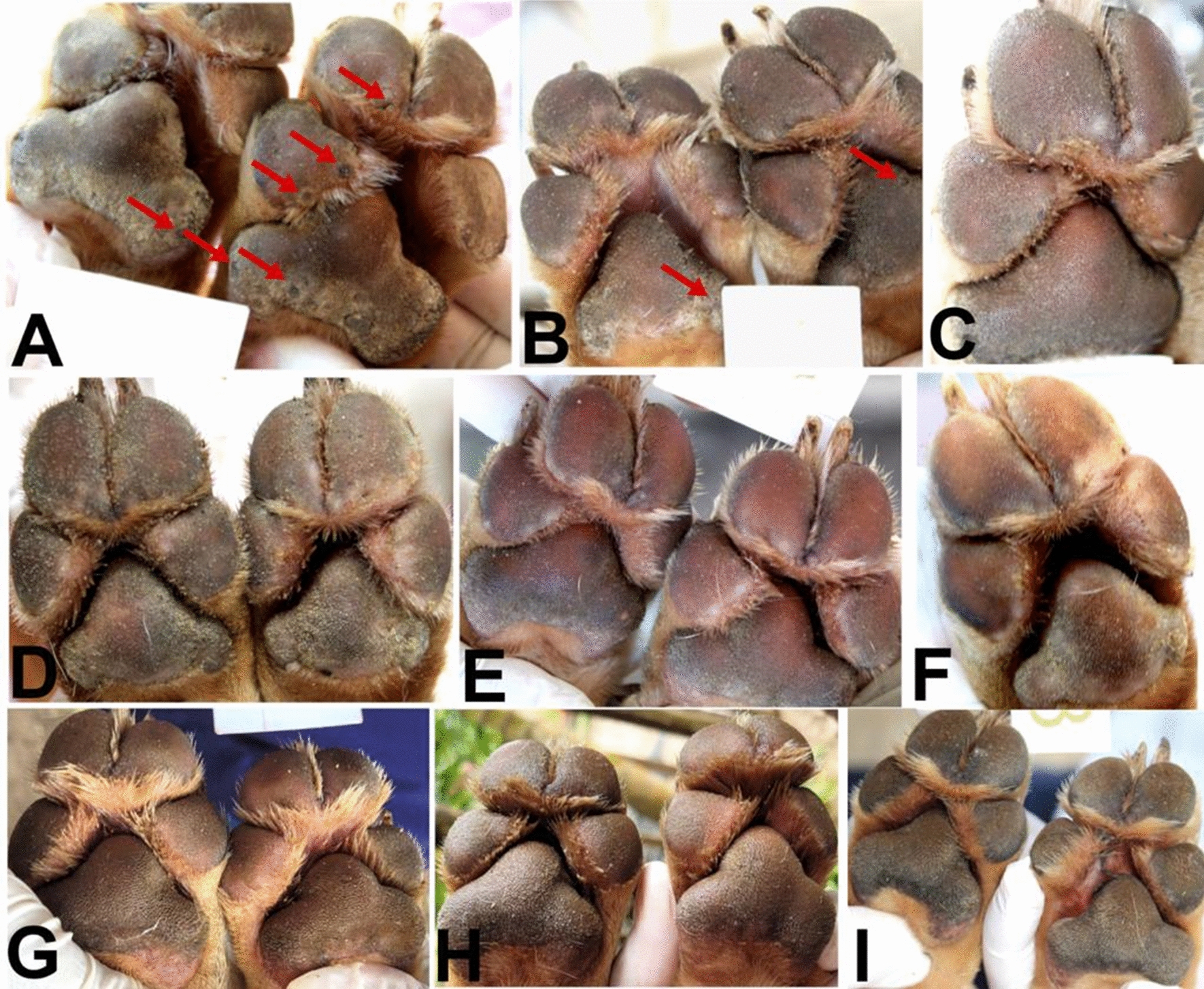
Evolution of *T. penetrans* lesions for dog 23 of the treated group (*source*: personal collection). **A** Day 0, the dog had multiple stage II and III lesions located on the front feet pads; **B**, **C** day 7, stage II and III lesions were no longer visible, stage V lesions were visible (arrows in **B**); **D** day 14 and **E**, **F** day 21, the pads remained free of stages II and III lesions; **G** day 30, **H** day 60, and **I** day 90, the pads were free of *T. penetrans* lesions and were completely re-epithelialized

**Fig. 4:**
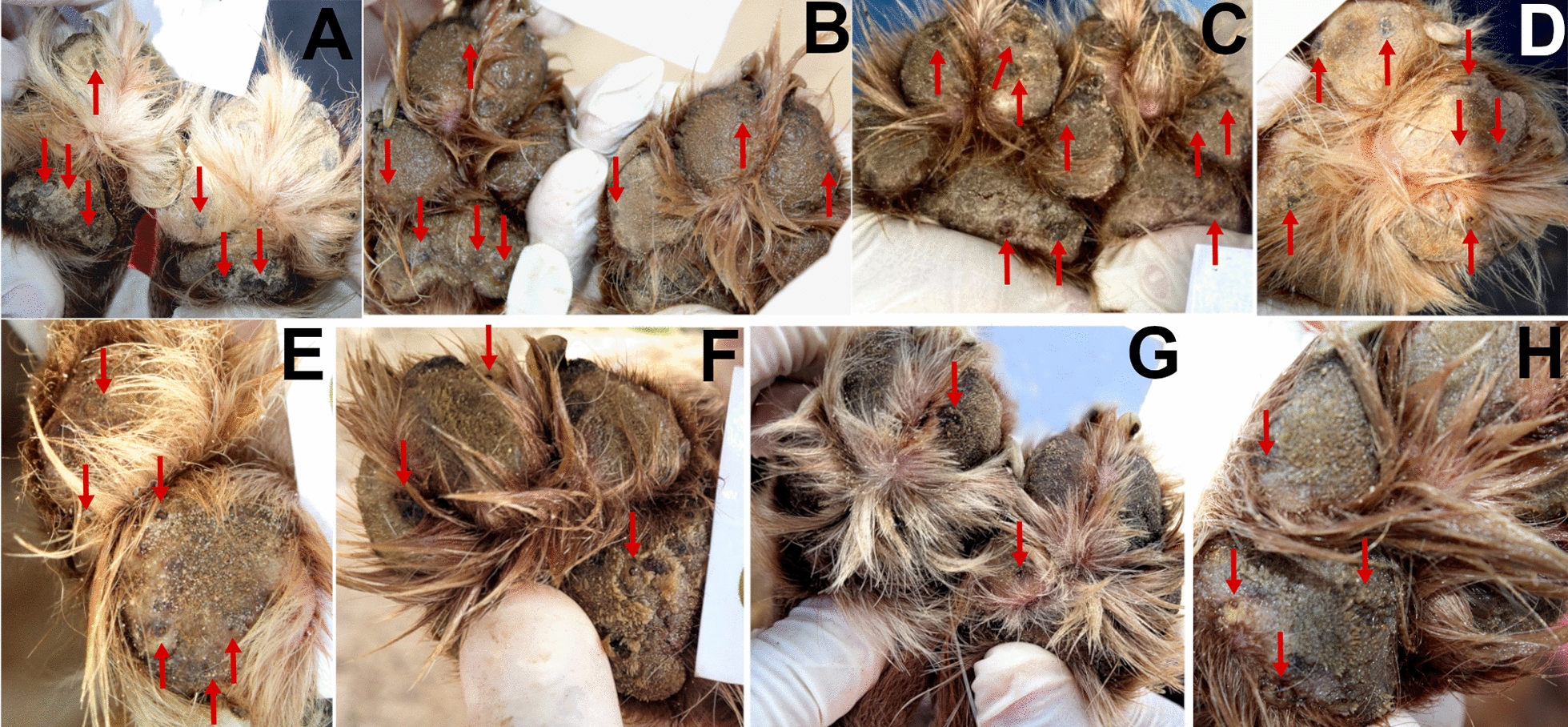
Evolution of *T. penetrans* lesions for dog 36 of the untreated control group (not co-living with a treated dog) (*source*: personal collection). **A** Day 0, **B** day 7, the dog had multiple stage II and III lesions located on the front feet pads; **C** day 14, in addition to the stage II and III lesions, stage IV lesions can be observed (arrows); **D**, **E** day 21, **F** day 30, **G** day 60, and **H** day 90, the pads remained infected with *T. penetrans* lesions

During phase I, even though significantly lower than in the IVP group, the number of *Tunga*-free dogs increased somewhat in the untreated control dogs, in particular 6 weeks (from day 44) after the first afoxolaner administration to the IVP-treated group (Table 4). The three monthly treatments with afoxolaner in an important proportion of the local dog population may have impacted the parasite turnover of the environmental contamination, thus reducing the infection rate in the untreated dogs. Figures 1 and 2 illustrate the evolution of *T. penetrans* lesions in a treated and an untreated control dog living in the same house yard. Figures 3 and 4 illustrate the evolution of *T. penetrans* lesions in a treated dog and an untreated control dog living alone in different house yards, respectively.

The most common treatment of tungiasis in humans is the mechanical extraction of the parasitic lesion followed by local/systemic symptomatic treatment of the inflammatory and secondary infectious consequences. Nevertheless, a sustained and efficient reduction in the local *Tunga* spp. prevalence in impoverished endemic areas requires drastic environmental control measures, which may be inefficient, difficult, and unaffordable in such areas [2, 11]. Besides the environmental hygienic measures and human–animal proximity controls, the direct treatment of *T. penetrans* in dogs, a major reservoir of *Tunga* spp., may contribute to the control of the disease in humans and provide an efficient one-health strategy to public health authorities [17, 28].

In the present study, afoxolaner provided a convenient and efficacious solution for the control of *T. penetrans* in dogs. NexGard Spectra^®^ is a highly palatable [40] chewable tablet and therefore easily accepted by dogs, which simplifies oral administration, including to individuals with a lower level of domestication that typically populate *T. penetrans* endemic areas. Besides palatability, which facilitates a high level of compliance, the monthly regimen of this product allows the treatment duration to be adapted in areas with seasonal peaks of infection.

NexGard Spectra^®^ combines afoxolaner and MO. The effect of MO on arthropods is believed to be negligible [39]; however, no specific data were obtained on its effect on embedded *T. penetrans.* It is possible that MO also contributed to the efficacy observed in this study, but only for a short duration after each IVP oral administration, because of its short half-life, i.e., 1.6 days ± 0.4 days for the A3 form and 3.3 days ± 1.4 days the A4 form [36, 38], and therefore in a negligible way relative to afoxolaner. Nevertheless, the decision was made to avoid the use of MO in the control group in this study, to avoid any confusion about the observations made on the untreated control animals. The dewormer used in the control group, a combination of pyrantel pamoate, fenbendazole, and praziquantel, did not have any active ingredient with a potential effect on *T. penetrans*.

Even though dogs are the main reservoir species of *T. penetrans*, the environment may be loaded with infective stages for a significant period, and other animal species may also play a reservoir role. Therefore, it would be highly valuable to further assess the correlation between human cases and the control of the parasite in dogs in endemic communities.

If confirmed, the obvious one-health benefit of the use of NexGard Spectra^®^ in dogs in relation to tungiasis may be further sustained by its nematicidal spectrum that includes several other zoonotic agents for which dogs are a reservoir [41]. For example, *Ancylostoma* spp. and *Toxocara* spp. are, through their larva migrans effect, another significant public health concern in many regions of the world including areas of tungiasis [42–45], and NexGard Spectra^®^ has registered efficacy against them [38].

## Conclusions

This study demonstrated that monthly oral administration of afoxolaner was highly effective for the treatment and control of tungiasis in dogs, the main reservoir of *T. penetrans* in many endemic areas in the world. Apart from an obvious beneficial effect on the health and welfare of the treated dog, the use of this product may also have a one-health benefit.

## Data Availability

All available data are within the manuscript.
